# Cardiovascular Magnetic Resonance Imaging-Based Right Atrial Strain Analysis of Cardiac Amyloidosis

**DOI:** 10.3390/biomedicines10123004

**Published:** 2022-11-22

**Authors:** Jan Eckstein, Vanessa Sciacca, Hermann Körperich, Lech Paluszkiewicz, Elena Weise Valdés, Wolfgang Burchert, Muhammed Gerçek, Martin Farr, Philipp Sommer, Christian Sohns, Misagh Piran

**Affiliations:** 1 Institute for Radiology, Nuclear Medicine and Molecular Imaging, Heart and Diabetes Center North Rhine Westphalia, University of Bochum, 32545 Bad Oeynhausen, Germany; 2 Clinic for Electrophysiology, Heart and Diabetes Center North Rhine Westphalia, University of Bochum, 32545 Bad Oeynhausen, Germany; 3 Clinic for Thoracic and Cardiovascular Surgery, Heart and Diabetes Center North-Rhine Westphalia, Ruhr-University of Bochum, 32545 Bad Oeynhausen, Germany; 4 Clinic for General and Interventional Cardiology, Heart and Diabetes Center North Rhine Westphalia, University of Bochum, 32545 Bad Oeynhausen, Germany; 5 Cardiogenetic Laboratory, Heart and Diabetes Center North-Rhine Westphalia, Ruhr-University of Bochum, 32545 Bad Oeynhausen, Germany

**Keywords:** cardiac strain, cardiac magnetic resonance imaging, cardiac amyloidosis, right atrium

## Abstract

**Background**: Cardiac amyloidosis (CA) manifests in a hypertrophic phenotype with a poor prognosis, making differentiation from hypertrophic cardiomyopathy (HCM) challenging and delaying early treatment. The extent to which magnetic resonance imaging (MRI) quantifies the right atrial strain (RAS) and strain rate (RASR), providing valuable diagnostic information, is not yet clinically established. **Aims**: This study assesses diagnostic differences in the longitudinal RAS and RASR between CA and HCM patients, control subjects (CTRL) and CA subtypes in addition to the impact of atrial fibrillation (AF) on the right atrial function in CA patients. The RAS and RASR of tricuspid regurgitation (TR) patients are used to assess the potential for diagnostic overlap. **Methods**: RAS and RASR quantification was conducted via MRI feature-tracking for biopsy-confirmed CA patients with subtypes identified. Strain parameters were compared for CTRL, HCM and TR patients. Post hoc testing identified intergroup differences. **Results**: In total, 41 CA patients were compared to 47 CTRL, 20 HCM and 31 TR patients. Reservoir (R), conduit and booster RAS and RASRs allow for significant differentiation (*p* < 0.001) between CA and HCM patients (R: 10.6 ± 14.3% vs. R: 33.5 ± 16.3%) and CTRL (R: 44.6 ± 15.7%). Booster and reservoir RAS and RASRs qualified as reliable diagnostic tests (AUC > 0.8). CA patients with AF, in contrast to sinus rhythm, demonstrated a significantly impaired reservoir RAS and RASR and booster RASR. The discriminative power of RAS for CA vs. TR was insufficient (R: 10.6% ± 14.3% vs. 7.0% ± 6.0%, *p* = 0.069). Differentiation between 21 transthyretin and 20 light-chain amyloidosis subtypes was not achievable (R: 0.7% ± 1.0% vs. 0.7% ± 1.0%, *p* = 0.827). **Conclusion**: The MRI-derived RAS and RASR are impaired in CA patients and may support noninvasive differentiation between CA, HCM and CTRL.

## 1. Introduction

Amyloidosis is a group of rare diseases characterized by the extracellular deposition of malfunctioned protein fibrils that distort tissue architecture, in turn affecting organic function [1]. Particularly, cardiac involvement is associated with increased mortality and morbidity [2]. The most common amyloidosis types are the acquired monoclonal immunoglobulin light-chain (AL) and transthyretin-related amyloidosis (ATTR) [3]. Regardless of the subtype, CA is a restrictive cardiomyopathy with diastolic dysfunction that can mimic hypertrophic cardiomyopathy (HCM) [4]. CA is most likely underdiagnosed, as a differential diagnosis to HCM remains clinically and radiologically challenging, but is of critical prognostic importance as treatment strategies between both diseases vary [5]. To date, the majority of cardiac strain studies have focused on the left ventricle [6,7,8,9] and left atrium [8,10,11], with most only comparing CA with control subjects (CTRL) [8,12]. Moreover, data exploring the amyloidotic impact on the right side of the heart remains scarce.

A recent echocardiographic study entailing 16 AL patients presented significantly reduced right atrial emptying fractions, increased right atrial volumes and impairment of the peak global and segmental strain compared to the CTRL [12]. These findings are suggestive of CA-dependent alterations in the right atrial strain (RAS) that may help distinguish between CA and HCM patients. In contrast to the left atrium, the right atrium may be less affected by HCM-associated mitral valve abnormalities [13]. Furthermore, HCM patients typically do not present atrial enlargement or atrial dysfunction [10], whereas CA commonly coincides with biatrial dilatation alongside interatrial septal thickening [2]. Thus, RAS may be a supportive tool for the differential diagnosis between CA and HCM.

A possible mimic of CA-associated RAS may be found in patients with tricuspid regurgitation (TR), as they often present dilatation and dysfunction of the right atrium [14]. Thus, the specificity of the CA-dependent RAS may be limited in its applicability, although the extent remains to be clarified.

To overcome the operator dependency associated with echocardiography and its limitations of the acoustic window [15], this study utilizes cardiovascular magnetic resonance imaging (CMR) for quantification of strain parameters. Recent studies have demonstrated the diagnostic value of late gadolinium enhancement (LGE) and T1 mapping as techniques to improve CA detection at both the ventricular and atrial levels [7,16,17,18]. However, the focus of this study was to exclusively evaluate the diagnostic value of the longitudinal RAS, RASR and right atrial volumetry in patients with CA and individual subtypes, including TR, HCM and CTRL subjects, in order to assess its noninvasive diagnostic value and limitations for a differential diagnosis between CA and HCM. Moreover, gadolinium administration is often contraindicated for amyloidotic patients, as renal insufficiency is not uncommon. Therefore, cardiac strain may exhibit potential as a noncontrast diagnostic support. As atrial fibrillation (AF) commonly affects CA patients [19,20], the impact of AF on right atrial strain parameters in CA patients was additionally analyzed.

## 2. Methods

### 2.1. Study Population

This is a retrospective, single-center, observational study. Inclusion criteria included biopsy-confirmed CA patients and their subtypes, along with adequate CMR quality. The time period of enrollment encompassed 2013–2020 for CA patients. CA patients with sinus rhythm (SR) and atrial fibrillation at the time of CMR examination were included and analyzed separately. CMR examination was performed according to standard diagnostic protocol.

### 2.2. Hypertrophic Cardiomyopathy Population

HCM patients were included upon confirmed diagnosis according to the 2014 ESC guidelines and 2020 AHA guidelines [21,22], and if they had received CMR. The time period of HCM patient enrollment spanned 2018–2021. HCM patients with AF during CMR examination were excluded.

### 2.3. Tricuspid Regurgitation Population

TR patients suffering from high-grade tricuspid valve insufficiency received a CMR examination prior to an elective catheter-based implantation of an annular reduction system. The time period of enrollment covered 2020–2021. All TR patients were included, despite the majority (94%) presenting with AF during CMR examination.

### 2.4. Healthy Control Subjects

Healthy volunteers served as CTRL. The time period of enrollment was 2017–2020. The group was aged over 50 years for an improved comparison with the study groups. Written informed consent was obtained from all participants. Exclusion criteria for CTRL subjects entailed a clinical history of cardiovascular disease and surgery, medication for cardiovascular or metabolic disorders, associated risk factors and contraindications for CMR. If CMR imaging demonstrated myocardial abnormalities, aortic ectasia, pulmonary trunk dilation, valvular heart disease, ischemic heart disease or signs of cardiomyopathy, the control individuals would be excluded. All examinations were carried out in accordance with the 1964 Declaration of Helsinki and the study was approved by the local ethics committee (Ethik-Kommission der Medizinischen Fakultät der Ruhr-Universität Bochum, Bochum, Germany; registration number 2017–238).

### 2.5. Clinical Parameters

Clinical data of patients were collected by consultation of medical records. This included the following data: age, gender, height, weight, NTproBNP, atrial fibrillation, diabetes mellitus, arterial hypertension, coronary heart disease, New York Heart Association (NYHA) score, chronic obstructive pulmonary disease (COPD) and stroke. The Institutional Ethical Review Board of the Ruhr University Bochum approved the retrospective data analysis and waived informed consent (Ethik-Kommission der Medizinischen Fakultät der Ruhr-Universität Bochum; registration number 2016-96-RDA-EV).

### 2.6. Cardiac MRI

All subjects underwent cardiac MRI at our institution using a 3.0 Tesla multi-transmit magnetic resonance imaging system (Achieva, Philips Healthcare, Best, the Netherlands; Release 5.3.1 and 5.6.1), incorporating dStream technology. Vector electrocardiogram-triggered cardiac cine acquisitions were performed in all patients. The maximum gradient performance was 40 mT/m with a slew rate of 200 mT/m/ms. A cardiac phased-array coil was used for signal reception. An axially acquired stack covering the whole heart and a short-axis stack covering the entirety of the left and right ventricles (12–16 slices, no gap) were utilized with retrospectively gated cine steady-state free-precession acquisitions (TR/TE/flip angle = 2.7 ms/1.35 ms/42°) for the assessment of heart function in all four cardiac chambers and morphology. A parallel imaging technique with a SENSE-reduction factor of 2 was applied to keep breath-holding times ≤12 s. Right atrial longitudinal strain and strain rate were assessed by applying 4-chamber, long-axis views. Within one cardiac cycle, >25 reconstructed heart frames were acquired in order to achieve a greater temporal resolution, as recently demonstrated [23]. At a typical heart rate of 70 beats per minute, the temporal resolution was 34 ms per cardiac frame. The spatial resolution was 1.5 × 1.5 × 8 mm^3^.

### 2.7. Strain Analysis

**Figure 1:**
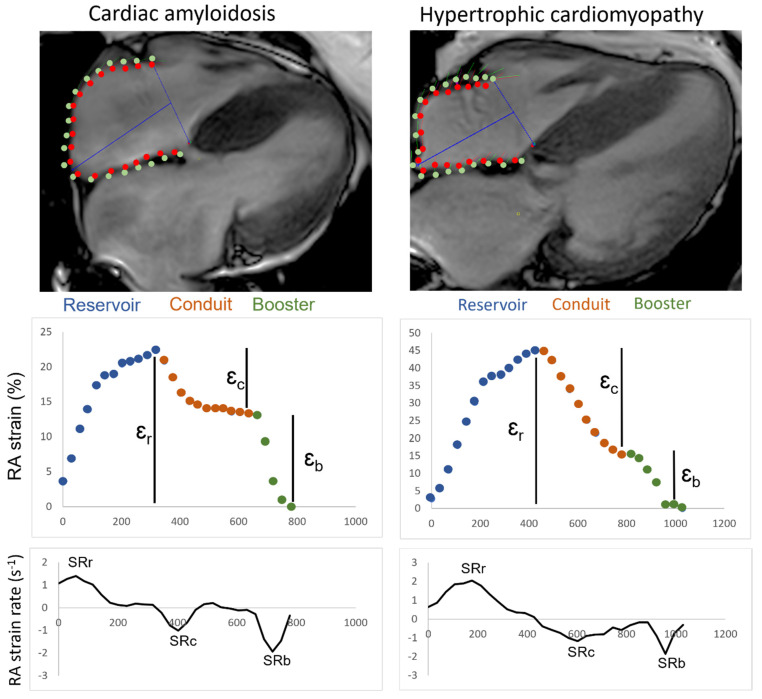
CMR Strain Analysis of CA and HCM. Contouring of the endocardial and epicardial right atrium in four-chamber view of a CA and HCM patient, along with corresponding strain and strain rate curves. RA—right atrial; ɛ_r_—reservoir strain; ɛ_c_—conduit strain; ɛ_b_—booster strain; SRr—reservoir strain rate; SRc—conduit strain rate; SRb—booster strain rate.

The longitudinal axis of the four-chamber view served as the quantitative parameter for RAS assessment. Strain analysis was conducted using the CVI42^®^ software package (Circle Cardiovascular Imaging Inc., Calgary, AB, Canada, Release 5.12.1) based on the cine steady-state free-precession acquisitions. Right atrial feature-tracking techniques for RAS and RASR quantification were applied as previously described [15]. At the end-diastole, contours of the right atrial endo- and epicardium were delineated manually in 4-chamber, long-axis slices (Figure 1). Using end-diastole over the onset of atrial contraction as the zero reference point is advantageous as it can be applied to patients with sinus rhythm or atrial fibrillation, as described recently [24]. These technical adjustments are of particular relevance considering patients with AF in the CA and TR patient cohort were included. The linings excluded the superior and inferior ostia of the vena cava, as well as the right atrial appendage. The global longitudinal RAS and RASR were then quantified for all patients and subjects. Reservoir, conduit and booster functions for each patient or subject were analyzed. Volumetric right atrial and ventricular quantifications were obtained using the Simpson approach at the end of diastole and systole.

### 2.8. Statistics

Statistical analysis was performed using SPSS (version 27.0.0.0, IBM Deutschland GmbH, Armonk, NY, USA). Continuous variables were presented as means ± standard deviations (SDs) when normally distributed; otherwise, as medians with interquartile ranges. The sample size calculation was carried out according to Grothues and colleagues [25], assuming a power of 90% and an alpha error of 0.05. The comparison of baseline characteristics, volumetric parameters and cardiac strain between four different groups was carried out using a univariate ANOVA or ANOVA-Welch if criteria were met. A post hoc Tukey-HSD test was conducted in the case of homogeneity of variance; otherwise, a Games–Howell test was used to identify intergroup differences. In case of nonparametric data, the Kruskal–Wallis test was used. The comparison between amyloidosis subtypes was carried out using an independent *t*-test for parametric datasets or the Mann–Whitney U test for a nonparametric dataset. Inter- and intraobserver variability were tested via Bland–Altman analyses, intraclass correlation coefficients (ICC, two-way mixed model, absolute agreement) and coefficients of variation (CoV). Strain and strain rate differences were presented as box plot graphs. Additionally, the sensitivity and specificity of strain data between CA and HCM patients were analyzed utilizing the receiver operating characteristic (ROC) analysis. The area under the curve (AUC) ranges were defined as 0.9–0.99 as an excellent test, 0.8–0.89 as a good test, 0.7–0.79 as a fair test and <0.7 as a non-useful test [26].

## 3. Results

### 3.1. Baseline Characteristics

This study includes 41 CA patients, 20 HCM patients, 31 TR patients and 47 CTRL subjects, with details summarized in Table 1. TR patients represent the oldest patients (82.0 ± 4.3 years), whereas CTRL subjects are the youngest (55.9 ± 10.4 years). The mean maximum indexed volumes indicate significantly increased right atrial dilatation in TR patients compared to CA patients (119.4 ± 41.6 mL/m^2^ vs. 48.0 ± 17.0 mL/m^2^, *p* < 0.001). Additionally, TR patients showed greater impairment of the right atrial ejection fraction compared to CA patients (30.0 ± 20.0% vs. 14.3 ± 11.0%, *p* < 0.001). CA patients differ significantly from HCM patients in all right atrial volumetric aspects. Volumetric data of the right ventricle in CA patients differ significantly for stroke, systolic and diastolic volume compared to CTRL and HCM patients; however, they do not differ significantly with regard to TR patients. Furthermore, the right ventricular ejection fraction is significantly impaired in CA patients compared to TR patients (36.4 ± 13.4% vs. 50.5 ± 8.4%, *p* < 0.001).

### 3.2. Right Atrial Strain and Strain Rate

**Figure 2:**
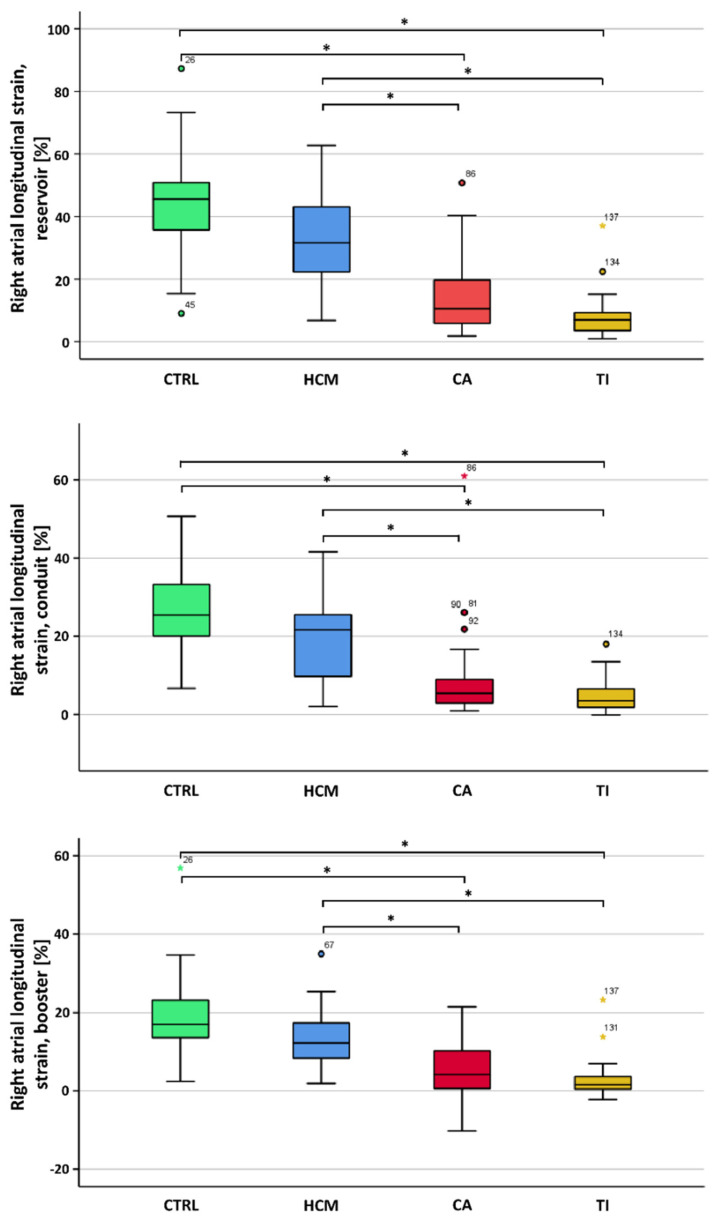
Box plots of right atrial longitudinal strain for functional phases of reservoir, conduit and booster between CTRL subjects and HCM, CA and TR patients. * Statistical significance.

Reservoir longitudinal RAS was significantly lowered in CA compared to HCM and CTRL (10.6 ± 14.3% vs. 33.5 ± 16.3% vs. 44.6 ± 15.7%, *p* < 0.001). Observations were consistent for conduit and booster RAS (Figure 2), as well as the reservoir, conduit and booster longitudinal RASR (Figure S1). Longitudinal RAS provided no significant differentiation between CA and TR patients. However, a significant difference between CA and TR patients was observed for the reservoir, conduit and booster RASR, with a maximum significance level at the booster phase (*p* = 0.001). Further details are summarized in Table 1.

### 3.3. Sensitivity and Specificity of RAS and RASR for HCM and CA Patients

**Figure 3:**
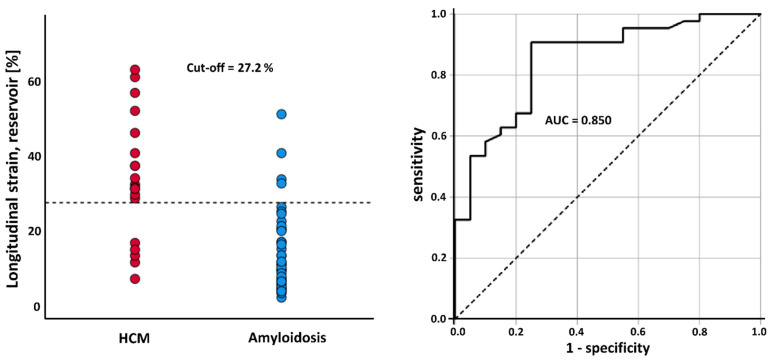
ROC analysis of booster longitudinal strain rate between HCM patients and CA patients.

ROC analyses found the conduit RAS and RASR as “fair” diagnostic tests (AUC = 0.793, 0.795). The reservoir and booster RAS and RASR presented as “good” diagnostic tests, with maximum sensitivity and specificity achieved with the booster RASR (AUC = 0.862) (Figure 3 and Table 2).

### 3.4. CA Patients with and without AF

Among the 41 CA patients, 17 (41.5%) CA patients had AF during CMR examination, correlating with their medical record (Table 3). There were greater numerical levels of NTproBNP (3465 ± 5938 vs. 2738 ± 3856) and a lowered incidence of stroke (1 vs. 4) for AF than SR, which were without statistical significance. Strain parameters of CA patients with AF showed a significant reduction in reservoir RAS (6.1 vs. 14, *p* = 0.007), reservoir RASR (0.4 vs. 0.9, *p* = 0.008) and booster RASR (−0.6 vs. −1.2, *p* = 0.013) compared to CA patients with SR.

### 3.5. Comparison of Subtypes ATTR and AL

From the volumetric data of both subtypes, no significant difference was found for the right atrium and ventricle. Further functional phases of the RAS and RASR provided no significant differentiation between the subtypes of ATTR and AL (Table S2).

### 3.6. Intra- and Interobserver Variation

Intra- and interobserver variability were tested on 20 randomized CA patients (Table 4). Intra- and interobserver variability resulted in excellent intraclass-correlation coefficients (ICC > 0.9) and a low coefficient of variation for intra- and interobserver variability (CoV < 10 %).

## 4. Discussion

To the best of our knowledge, this study is the first to provide a systematic cardiac MRI assessment of RAS and RASR for CA patients. Differentiating CA from HCM is an ongoing challenge. However, the volumetric parameters, RAS and RASR may offer discriminative features with diagnostic potential. The following novel observations were made throughout this study:

I. Right atrial volumetric data significantly differ between CA patients and all other groups.
II. RAS and RASR reflect significant impairment in CA patients with and without AF versus HCM and CTRL patients. However, RAS is insufficient for discrimination of CA from TR.
III. Strain and volumetric data provide no discrimination between amyloidotic subtypes.
IV. AF exacerbates the right atrial function in CA patients.

### 4.1. The Diagnostic Value of Right Atrial Volumetric Data

To the best of our knowledge, our study is the first cardiac MRI study to share consistent observations of recent echocardiographic data [12]. Right atrial volumetric data appear to provide discriminatory features for the aspects of the right atrial dilation and right atrial ejection fraction, which are valuable for CA characterization. Although the mean maximum indexed right atrial volume of CA patients lies in the upper margin and the mean right atrial ejection fraction in the lower margin of the reference range of healthy subjects [27], they differ significantly from those of HCM, TR and CTRL subjects. A previous echocardiographic study shared similar findings by observing left atrial enlargement prior to left ventricular wall thickening or left ventricular systolic impairment [28]. Their observation was consolidated by Higashi and colleagues, who found the left atrial dilatation grade and left atrial emptying fraction to be a “good” diagnostic test after ROC analyses in CA patients [10]. Protein fibril infiltration of the atrial chambers appears to provide MRI-quantifiable volumetric alterations with diagnostic value.

### 4.2. The Diagnostic Value of MRI-Based Strain Data

Nemes and colleagues did not observe differences in the longitudinal global or longitudinal mean segmental strain of CA compared to CTRL subjects. Instead, their study found significant regional circumferential strain and longitudinal strain differences of the right atrium in CA compared to CTRL patients [12]. Although 3D speckle-tracking echocardiography (STE) was utilized in their study, intramodality differences cannot be ruled out and may explain observational differences. In our study, CA patients presented significantly reduced longitudinal RAS and RASRs in all functional phases compared to HCM and CTRL patients, mirroring the severe impairment of cardiac deformation. Additionally, we observed good specificity and sensitivity of the reservoir and booster RAS and RASR for differentiation between HCM and CA patients. Interestingly, the conduit RAS and RASR only presented as a “fair” diagnostic test, presumably because this functional phase is most passive.

### 4.3. CA versus TR Patients

A recent echocardiographic study observed increased RA stiffness with increasing TR severity, significantly reducing RA reservoir-phase mechanics [29]. Our strain and volumetric data demonstrate the mechanical impairment of the right atrium throughout severe TR. Moreover, the right atrial dilatation of TR patients significantly exceeded that of CA patients. However, reservoir, conduit and booster RAS could not discriminate the right atrial impairment of TR patients from that of CA patients. This could only be observed for the RASR. Although the MRI phenotype of TR patients, especially in its severe form, can usually be distinguished from patients with a hypertrophic phenotype, our study hereby presents the diagnostic limitations of RAS.

### 4.4. AL versus ATTR Subtypes

Subtypes of CA have been distinguished utilizing transmural LGE patterns [6] and electrocardiographic, echocardiographic and hemodynamic differences [3]. Differences between subtypes were argued to be the result of amyloid deposition frequency [3,5,30]. Despite demonstrating a metric with an absence of volumetric differences, our study observed no correlative functional differences in regard to volumetric and strain data between AL and ATTR patients. Our findings are not in line with the recent results of Palmer and colleagues, who observed a significantly greater impairment of left atrial reservoir and booster strain for ATTR compared to the AL subtype [31]. However, the classification of CA disease staging is still undergoing continuous refinement [32,33] and undoubtedly remains a challenge, especially when attempting to compare between CA subtypes. Thus, it may be that advanced stages of CA disease are responsible for the absence of differences in CMR strain parameters between CA subtypes.

### 4.5. CA Patients with and without AF

The observations of this study are in line with recent findings that present AF as a common comorbidity in CA with a greater incidence among the ATTR than the AL subtype [19,20]. The increased stroke incidence in CA patients in SR is most likely associated with left atrial dysfunction, as recently described [8,10,11]. A recent study on stroke patients suggested that atrial premature complexes may be associated with LA dilatation and dysfunction, which could lead to cardiac embolism even in absence of AF [34]. These pathophysiological changes may apply to amyloidotic cardiomyopathy.

A further novel finding of this study is the elevated impairment of the reservoir RAS and RASR, along with the booster RASR for CA patients with AF in contrast to the SR. Therefore, AF appears to exacerbate right atrial function despite underlying amyloid infiltration. Consistent with recent observations regarding the left atrium, the booster function did not increase to compensate for the impaired reservoir function [31].

### 4.6. Clinical Implications of RAS and RASR

Among CMR, myocardial scintigraphy and light-chain assessments are effective noninvasive tools for CA diagnostics. Nonetheless, a relevant number of patients still receive an endocardial biopsy to confirm diagnosis. Confirmation of CA diagnostics commonly requires invasive myocardial biopsy. The discriminative value of T1 mapping and LGE has already been demonstrated [7,16,17,18]. The focus of this study was devoted to the assessment of the diagnostic value of the RAS, RASR and corresponding volumetrics. However, limiting diagnostics to single parameters entails the risk of diagnosing false positives. For example, in our study, TR patients could mimic CA patients using RAS due to a similar impairment of longitudinal deformation. Although TR patients present clinically and phenotypically different, it is important that diagnostic overlaps are avoided. However, integrating additional parameters, such as indexed right atrial volumes and the ejection fraction, may provide greater discriminative power. Thus, utilizing a multiparametric MRI approach offers promising developments in this aspect. Our findings may optimize noninvasive CMR diagnostics through the incorporation of RAS and RASR.

### 4.7. Intra- and Interobserver Variation

A high resemblance in intra- and interobserver measurements reflects the reliability of right atrial strain quantification using CMR, particularly for RAS. Although recent CMR findings have shown superior reproducibility of the left rather than the right atrium [35], our study demonstrates the potential role of CMR for right atrial strain quantification.

### 4.8. Limitations

This study is a retrospective, single-center, observational design with typical limitations. Due to retrospective image processing, quantification of the right atrium included only 4-chamber cine data, as routine diagnostic protocols were followed for CMR examinations of CA and HCM patients. A correction for multiple testing was not performed. It must be acknowledged that RA strain parameters may have no additional diagnostic value beyond reference left ventricular tissue characterization as T1 mapping and ECV findings were not assessed due to retrospective data inconsistencies. Although cohorts were not matched, we attempted to provide greater homogeneity by only including healthy subjects above the age of 50 years. Furthermore, overlapping structural changes of hypertensive heart disease cannot be excluded. Nevertheless, this is the first MRI-based study to present right atrial volumetric and strain data for biopsy-validated CA patients.

## 5. Conclusions

Overall, we demonstrate novel aspects in MRI-attained RAS, RASR and volumetric data, which present a supportive radiological function with the potential of improving differential diagnostics between patients with CA and HCM. However, right atrial strain parameters remain insufficient for distinguishing TR from CA patients or for the differentiation of CA subtypes.

## Figures and Tables

**Table 1:** Baseline parameters of HCM, CA, TR and CTRL subjects.

	CTRL	HCM	CA	TR	Comparison	Global Test	Post Hoc Test
N	47	20	41	31			
Males (%)	25 (53%)	10 (50%)	32 (78%)	16 (52%)			
Atrial fibrillation (%)	-	1 (5.0)	17 (41.5)	29 (93.5)			
Arterial hypertension (%)	-	10 (50.0)	32 (78.0)	24 (77.4)			
Coronary artery disease (%)	-	2 (10.0)	13 (31.7)	9 (29.0)			
COPD (%)	-	1 (5.0)	3 (7.3)	3 (9.7)			
Diabetes mellitus (%)	-	3 (15.0)	1 (2.4)	8 (25.8)			
Stroke (%)	-	1 (5.0)	5 (12.2)	4 (12.9)			
NYHA (%)	-						
I		1 (5.0)	0 (0)	0 (0)			
II		8 (40.0)	6 (14.6)	8 (25.8)			
III		11 (55.0)	33 (80.5)	23 (74.2)			
IV		0 (0)	2 (4.9)	0 (0)			
Age (yrs) ^b^	55.9 ± 10.4 ^a^	63.9 ± 7.4	79 ± 14 ^a^	82 ± 4 ^a^	CTRL-HCM CTRL-CA CTRL-TR HCM-CA HCM-TI CA-TR	*p* < 0.001	*p* = 0.007 *p* < 0.001 *p* < 0.001 *p* < 0.001 *p* < 0.001 *p* = 0.072
Weight (kg) ^c^	74.8 ± 12.8	83.6 ± 81.5	76.3 ± 75	71.2 ± 11.1	CTRL-HCM CTRL-CA CTRL-TR HCM-CA HCM-TR CA-TR	*p* = 0.014	*p* = 0.064 *p* = 0.950 *p* = 0.645 *p* = 0.175 *p* = 0.008 *p* = 0.369
Height (cm) ^c^	172.7 ± 10.7	171.7 ± 9.2	173.4 ± 10.3	165.9 ± 9.9	CTRL-HCM CTRL-CA CTRL-TR HCM-CA HCM-TI CA-TR	*p* = 0.015	*p* = 0.985 *p* = 0.984 *p* = 0.029 *p* = 0.922 *p* = 0.209 *p* = 0.013
BSA (m²) ^c^	1.9 ± 0.2	2 ± 0.2	1.9 ± 0.2	1.8 ± 0.2	CTRL-HCM CTRL-CA CTRL-TR HCM-CA HCM-TR CA-TR	*p* = 0.027	*p* = 0.441 *p* = 0.985 *p* = 0.216 *p* = 0.624 *p* = 0.019 *p* = 0.125
BMI (kg/m²) ^d^	25.0 ± 2.7	28.3 ± 3.9	24.6 ± 3.9 ^a^	25.8 ± 3.3	CTRL-HCM CTRL-CA CTRL-TR HCM-CA HCM-TR CA-TR	*p* = 0.002	*p* < 0.001 *p* = 0.916 *p* = 0.224 *p* < 0.001 *p* = 0.026 *p* = 0.197
HR (bpm) ^d^	64.9 ± 9.7	63.1 ± 7.8	74.0 ± 14.8	71.5 ± 10.7	CTRL-HCM CTRL-CA CTRL-TR HCM-CA HCM-TR CA-TR	*p* < 0.001	*p* = 0.436 *p* = 0.001 *p* = 0.008 *p* = 0.001 *p* = 0.004 *p* = 0.671
RA Vmax_i_ (mL/m²) ^b^	41.5 ± 9.8	40.2 ± 6.8	48.0 ± 17.0 ^a^	119.4 ± 41.6	CTRL-HCM CTRL-CA CTRL-TR HCM-CA HCM-TR CA-TR	*p* < 0.001	*p* = 0.929 *p* = 0.014 *p* < 0.001 *p* = 0.004 *p* < 0.001 *p* < 0.001
RA Vmin_i_ (mL/m²) ^b^	21.0 ± 7.0 ^a^	14.5 ± 3.9	35.0 ± 14.5 ^a^	99.4 ± 36.9	CTRL-HCM CTRL-CA CTRL-TR HCM-CA HCM-TR CA-TR	*p* < 0.001	all *p* < 0.001
RA SV_i_ (mL/m²) ^b^	19.2 ± 7.3	25.7 ± 6.9	13.0 ± 12.0 ^a^	18.8 ± 16.5 ^a^	CTRL-HCM CTRL-CA CTRL-TR HCM-CA HCM-TR CA-TR	*p* < 0.001	*p* = 0.007 *p* = 0.164 *p* = 0.986 *p* < 0.001 *p* = 0.146 *p* = 0.325
RA EF (%) ^d^	48.39 ± 17.0 ^a^	63.4 ± 9.8	30.0 ± 20.0 ^a^	14.3 ± 11.0 ^a^	CTRL-HCM CTRL-CA CTRL-TR HCM-CA HCM-TR CA-TR	*p* < 0.001	*p* = 0.002 *p* = 0.002 *p* < 0.001 *p* < 0.001 *p* < 0.001 *p* < 0.001
RV EDV_i_ (mL/m²) ^b^	76.4 ± 12.7	70.4 ± 11.9	87.6 ± 20.2	102.1 ± 27.1	CTRL-HCM CTRL-CA CTRL-TR HCM-CA HCM-TR CA-TR	*p* < 0.001	*p* = 0.253 *p* = 0.014 *p* < 0.001 *p* < 0.001 *p* < 0.001 *p* = 0.074
RV ESV_i_ (mL/m²) ^b^	30.4 ± 8.2	30.9 ± 10.4	55.4 ± 18.1	50.6 ± 17.1	CTRL-HCM CTRL-CA CTRL-TR HCM-CA HCM-TR CA-TR	*p* < 0.001	*p* = 0.997 *p* < 0.001 *p* < 0.001 *p* < 0.001 *p* < 0.001 *p* = 0.661
RV SV_i_ (mL/m²) ^d^	46.1 ± 6.8	39.5 ± 11.7	32.0 ± 25.0 ^a^	51.5 ± 15.6	CTRL-HCM CTRL-CA CTRL-TR HCM-CA HCM-TR CA-TR	*p* < 0.001	*p* = 0.023 *p* < 0.001 *p* = 0.270 *p* = 0.117 *p* = 0.003 *p* < 0.001
RV EF (%) ^b^	60.7 ± 5.7	65.0 ± 13.0 ^a^	36.4 ± 13.4	50.5 ± 8.4	CTRL-HCM CTRL-CA CTRL-TR HCM-CA HCM-TR CA-TR	*p* < 0.001	*p* = 0.952 *p* < 0.001 *p* < 0.001 *p* < 0.001 *p* < 0.001 *p* < 0.001

^a^ median value; ^b^ ANOVA-Welch; ^c^ ANOVA; ^d^ Kruskal–Wallis test; CTRL—control subjects; HCM—hypertrophic cardiomyopathy patients; CA—cardiac amyloidosis patients; TR—tricuspid regurgitation patients; BSA—body surface area; BMI—body mass index; RA Vmaxi—indexed maximum right atrial volume; RA Vmini—indexed minimum right atrial volume; RA SVi—indexed right atrial stroke volume; RA EF—right atrial ejection fraction; RV EDVi—indexed right ventricular end-diastolic volume; RV ESVi—indexed right ventricular end-systolic volume; RV SVi—indexed right ventricular stroke volume; RV EF—right ventricular ejection fraction; LV EF—left ventricular ejection fraction.

**Table 2:** ROC analyses of RAS and RASR for differentiation of CA and HCM patients.

	Cut-Off Value	AUC	Quality	Sensitivity	Specificity
Right atrial strain					
Reservoir (%)	27.2	0.850 (0.749 to 0.951)	good	90.7	75.0
Conduit (%)	12.2	0.793 (0.664 to 0.922)	fair	81.4	75.0
Booster (%)	7.4	0.804 (0.697 to 0.911)	good	62.8	90.0
Right atrial strain rate					
Reservoir (s^−1^)	0.85	0.829 (0.730 to 0.928)	good	60.5	95.0
Conduit (s^−1^)	−0.75	0.759 (0.615 to 0.903)	fair	81.4	65.0
Booster (s^−1^)	−1.45	0.862 (0.761 to 0.962)	good	79.1	90.0

AUC—area under the curve; CA—cardiac amyloidosis patients; HCM—hypertrophic cardiomyopathy patients.

**Table 3:** CA patients with and without atrial fibrillation.

	SR	AF	*p*-Value
N (%)	24 (58.5)	17 (41.4)	
Arterial hypertension (%)	16 (66.7)	16 (94.1)	*p* = 0.039
NTproBNP (ng/L)	2551 ± 3766	3465 ± 5938	*p* = 0.223
Coronary artery disease (%)	4 (16.7)	9 (52.9)	*p* = 0.015
COPD (%)	1 (4.2)	2 (11.8)	*p* = 0.363
Diabetes mellitus (%)	0	1 (5.9)	*p* = 0.235
Stroke (%)	4 (16.7)	1 (5.9)	*p* = 0.304
NYHA (%)			
I	0	0	
II	4 (16.7)	2 (11.8)	
III	20 (83.3)	13 (76.5)	
IV	0	2 (11.8)	
Right atrial strain			
Reservoir (%) ^b^	14.0 {12.6}	6.1 {7.0}	*p* = 0.007
Conduit (%) ^b^	6.0 {10.6}	4.3 {4.5}	*p* = 0.109
Booster (%) ^b^	7.4 {10.7}	1.7 {4.4}	*p* = 0.059
Right atrial strain rate			
Reservoir (s^−1^) ^b^	0.90 {1.25}	0.40 {0.75}	*p* = 0.008
Conduit (s^−1^) ^b^	−0.50 {0.35}	−0.40 {0.30}	*p* = 0.089
Booster (s^−1^) ^b^	−1.20 {0.80}	−0.60 {0.65}	*p* = 0.013

Median value {interquartile range}; ^b^ Mann–Whitney U test; SR—sinus rhythm; AF—atrial fibrillation.

**Table 4:** Comparison of CA patients vs. HCM patients with and without atrial fibrillation and impact on right atrial global longitudinal strain.

	CA	HCM	*p*-Value
*CA Without AF*			
Right atrial strain			
Reservoir (%) ^b^	16.1 {14.0}	34.0 {18.8}	*p* = 0.001
Conduit (%) ^b^	8.7 {10.8}	23.0 {14.1}	*p* = 0.004
Booster (%) ^b^	8.0 {12.3}	11.6 {10.1}	*p* = 0.027
Right atrial strain rate			
Reservoir (s^−1^) ^b^	1.20 {1.18}	2.00 {1.40}	*p* = 0.007
Conduit (s^−1^) ^b^	−0.60 {0.45}	−1.30 {1.10}	*p* = 0.039
Booster (s^−1^) ^b^	−1.15 {0.85}	−1.90 {0.80}	*p* < 0.001
*CA With AF ^†^*			
Right atrial strain			
Reservoir (%) ^b^	7.0 {6.3}	34.0 {18.8}	*p* < 0.001
Conduit (%) ^b^	3.7 {4.8}	23.0 {14.1}	*p* < 0.001
Booster (%) ^b^	2.3 {4.8}	11.6 {10.1}	*p* < 0.001
Right atrial strain rate			
Reservoir (s^−1^) ^b^	0.40 {0.65}	2.00 {1.40}	*p* < 0.001
Conduit (s^−1^) ^b^	−0.30 {0.40}	−1.30 {1.10}	*p* = 0.001
Booster (s^−1^) ^b^	−0.60 {0.90}	−1.90 {0.80}	*p* < 0.001

Median value {interquartile range}; ^b^ Mann–Whitney U test; † comparison of CA patients with AF vs. HCM patients without AF; AF—atrial fibrillation; CA—cardiac amyloidosis; HCM—hypertrophic cardiomyopathy.

## Data Availability

The data that support the findings of this study are available on request from the corresponding author. The data are not publicly available due to restrictions as it is containing information that could compromise the privacy of research participants.

## Supplementary Materials

The following supporting information can be downloaded at: https://www.mdpi.com/article/10.3390/biomedicines10123004/s1, Table S1: Right atrial strain and strain rates of CA-, HCM-, TR-patients and CTRL. Table S2: Differentiation between ATTR and AL subtypes of CA. Table S3: Percent interobserver and intraobserver variability of right atrial strain and strain rate.
