# Supplementary material for: Cardiovascular Magnetic Resonance Imaging-Based Right Atrial Strain Analysis of Cardiac Amyloidosis
Source: Biomedicines. 2022 Nov 22;10(12):3004. doi: 10.3390/biomedicines10123004 (PMC9775378; doi:10.3390/biomedicines10123004)
Supplement: Supplementary file 1 [file biomedicines-10-03004-s001.zip › biomedicines-1885759-supplementary.pdf]

Supplement Data

Table S1. Right atrial strain and strain rates of CA-, HCM-, TR-patients and CTRL.

	Control	HCM	CA	TR	Comparison	Global test	Post-Hoc Test
right atrial strain							
reservoir [%][d]	44.6 ± 15.7	33.5 ± 16.3	10.6 ± 14.3[a]	7.0 ± 6.0[a]	CTRL-HCM<br>CTRL-CA<br>CTRL-TR<br>HCM-CA<br>HCM-TR<br>CA-TR	$p < 0.001$	$p < 0.081$<br>$p < 0.001$<br>$p < 0.001$<br>$p < 0.001$<br>$p < 0.001$<br>$p = 0.069$
conduit [%][d]	25.7 ± 10.1	19.9 ± 11.7	5.4 ± 6.4[a]	3.5 ± 5.1[a]	CTRL-HCM<br>CTRL-CA<br>CTRL-TR<br>HCM-CA<br>HCM-TR<br>CA-TR	$p < 0.001$	$p < 0.106$<br>$p < 0.001$<br>$p < 0.001$<br>$p < 0.001$<br>$p < 0.001$<br>$p = 0.146$
booster [%][d]	17 ± 10.3[a]	13.6 ± 7.7	5.3 ± 6.5	1.6 ± 3.3[a]	CTRL-HCM<br>CTRL-CA<br>CTRL-TR<br>HCM-CA<br>HCM-TR<br>CA-TR	$p < 0.001$	$p < 0.069$<br>$p < 0.001$<br>$p < 0.001$<br>$p = 0.001$<br>$p < 0.001$<br>$p = 0.153$
right atrial strain rate							
reservoir [s^{-1}][d]	2.2 ± 1.1[a]	2 ± 1.5[a]	0.7 ± 1.1[a]	0.2 ± 0.5[a]	CTRL-HCM<br>CTRL-CA<br>CTRL-TR<br>HCM-CA<br>HCM-TR<br>CA-TR	$p < 0.001$	$p = 0.379$<br>$p < 0.001$<br>$p < 0.001$<br>$p < 0.001$<br>$p < 0.001$<br>$p = 0.013$
conduit [s^{-1}][d]	−2.2 ± 1	−1.2 ± 0.8	−0.5 ± 0.5[a]	0.1 ± 0.6[a]	CTRL-HCM<br>CTRL-CA<br>CTRL-TR<br>HCM-CA<br>HCM-TR<br>CA-TR	$p < 0.001$	$p = 0.003$<br>$p < 0.001$<br>$p < 0.001$<br>$p = 0.016$<br>$p < 0.001$<br>$p = 0.012$
booster [s^{-1}][d]	−2.4 ± 1.0	−2.1 ± 0.9	−0.8 ± 0.9[a]	−0.2 ± 0.4[a]	CTRL-HCM<br>CTRL-CA<br>CTRL-TR<br>HCM-CA<br>HCM-TR<br>CA-TR	$p < 0.001$	$p = 0.579$<br>$p < 0.001$<br>$p < 0.001$<br>$p < 0.001$<br>$p < 0.001$<br>$p = 0.001$

[a] – median value, [b] - ANOVA-Welch, [c] - CTRL – ANOVA, [d] - Kruskal -Wallis –Test, CTRL – control subjects, HCM – hypertrophic cardiomyopathy patients, CA – cardiac amyloidosis patients, TR- tricuspid regurgitation patients.

Table S2. Differentiation between ATTR and AL subtypes of CA.

	ATTR	AL	P-Value
N	21	20	
Atrial fibrillation (%)	10 (47.6)	7 (35.0)	$p = 0.418$
Arterial hypertension (%)	18 (85.7)	14 (70.0)	$p = 0.230$
NTproBNP [ng/l]	2278 ± 3262	3615 ± 5927	$p = 0.744$
Coronary artery disease (%)	11 (52.4)	2 (10.0)	$p = 0.004$
COPD (%)	2 (9.5)	1 (5.0)	$p = 0.583$
Diabetes mellitus (%)	0 (0)	1 (5.0)	$p = 0.306$
Stroke (%)	1 (4.8)	4 (20.0)	$p = 0.141$
NYHA (%)			
I	0 (0)	0 (0)	
II	3 (14.3)	3 (15.0)	
III	17 (81.0)	16 (80.0)	
IV	1 (4.8)	1 (5.0)	
right atrial volumetrics			
RA $Vmax_i$ [ml/m²][b]	52.2 ± 14	46.8 ± 13.5	$p = 0.213$
RA $Vmin_i$ [ml/m²][c]	35.8 ± 14.2	31.6 ± 13.6	$p = 0.457$
RA SV_i [ml/m²][b]	13 ± 11.5[a]	13.5 ± 14.3[a]	$p = 0.604$
RA EF [%][b]	27.9 ± 19[a]	30.2 ± 24[a]	$p = 0.725$
right ventricular volumetrics			
RV EDV_i [ml/m²][b]	87.5 ± 21.1	87.7 ± 19.8	$p = 0.985$
RV ESV_i [ml/m²][b]	55.3 ± 17.9	55.5 ± 18.6	$p = 0.978$
RV SV_i [ml/m²][b]	32.2 ± 12.9	32.2 ± 12.6	$p = 0.993$
RV EF [%][b]	36.3 ± 13.2	36.4 ± 13.9	$p = 0.988$
right atrial strain			
reservoir [%][b]	9.5 ± 14.3[a]	10.3 ± 14.1[a]	$p = 0.966$
conduit [%][c]	5.5 ± 4.6[a]	4.9 ± 8.8[a]	$p = 0.840$
booster [%][b]	6.5 ± 6.2	4.7 ± 6	$p = 0.347$
right atrial strain rate			
reservoir [s^{-1}][b]	0.7 ± 1.0[a]	0.7 ± 1.0[a]	$p = 0.827$
conduit [s^{-1}][b]	−0.4 ± 0.2	−0.5 ± 0.7[a]	$p = 0.144$
booster [s^{-1}][b]	−0.7 ± 0.9[a]	−1.1 ± 0.6	$p = 0.322$

[a] – median value, [b] – Unpaired t-test, [c] – Mann-Whitney U-Test, ATTR – transthyretin related amyloidosis, AL – light chain amyloidosis, RA $Vmax_i$ – indexed maximum right atrial volume, RA $Vmin_i$ – indexed minimum right atrial volume, RA SV_i – indexed right atrial stroke volume, RA EF - right atrial ejection fraction, RV EDV_i – indexed right ventricular end diastolic volume, RV ESV_i – indexed right ventricular end systolic volume, RV SV_i – indexed right ventricular stroke volume, RV EF - right ventricular ejection fraction.

Table S3. Percent interobserver and intraobserver variability of right atrial strain and strain rate.

	right atrial strain			right atrial strain rate		
	**reservoir**	**conduit**	**booster**	**reservoir**	**conduit**	**booster**
intraobserver						
mean ± SD [%]	3.4 ± 7.4	11.5 ± 21.1	3.3 ± 13.2	−5.2 ± 12.9	−4.8 ± 18.6	6.0 ± 17.8
LOA [%]	18.2 to −11.4	53.6 to −30.7	29.8 to −23.2	20.6 to −31.0	32.3 to −42.0	41.5 to −29.5
ICC [%]	0.997	0.998	0.996	0.992	0.984	0.983
CoV [%]	4.5	9.9	4.0	6.8	−8.8	−6.8
interobserver						
mean ± SD [%]	4.5 ± 6.2	8.9 ± 21.6	8.8 ± 18.0	3.2 ± 12.9	−10.8 ± 18.6	−3.3 ± 13.8
LOA [%]	17.0 to −8.0	52.1 to −34.3	44.8 to −27.3	29.0 to −22.5	26.5 to −48.0	24.3 to −30.9
ICC [%]	0.997	0.997	0.997	0.964	0.991	0.994
CoV [%]	4.6	11.8	2.8	6.7	−6.9	−7.5

SD – standard deviation, LOA – limits of agreement, ICC – intra-class-correlation coefficients, CoV- coefficient of variation.
